# Evaluating the Ascertainment Bias of SARS-CoV-2 Using Spatial Backcalculation of Airport Screening Data in Japan

**DOI:** 10.3390/idr18050117

**Published:** 2026-10-09

**Authors:** Shiqi Liu, Asami Anzai, Hiroshi Nishiura

**Affiliations:** 1 School of Public Health, Graduate School of Medicine, Kyoto University, Yoshida Konoe Cho, Sakyo-ku, Kyoto 606-8501, Japan; 2 Department of Epidemiology, Japan Institute for Health Security, 1-21-1 Toyama, Shinjuku-ku, Tokyo 162-8655, Japan; 3 Center for Health Security, Graduate School of Medicine, Kyoto University, Yoshida Konoe Cho, Sakyo-ku, Kyoto 606-8501, Japan; 4 Department of Epidemiology and Biostatistics, Schulich School of Medicine & Dentistry, Western University, 1465 Richmond Street, London, ON N6G 2M1, Canada

**Keywords:** diagnosis, border control, quarantine, travel, statistical estimation, surveillance

## Abstract

Background: Despite global efforts to strengthen surveillance systems, ascertainment bias persisted. We evaluated the extent of this bias by comparing Japan’s airport entry screening data with local surveillance data. Methods: Entry screening data of non-Japanese passengers arriving from 25 countries were analyzed, and countries were categorized by income level: high-income countries (HICs), upper-middle-income countries (UMICs), and lower-middle-income countries (LMICs). A statistical model was developed to describe entry screening positivity using surveillance data of the country of origin. Results: Ascertainment bias was lowest in HICs (positivity = 1.76 times that of local surveillance data, 95% confidence interval [CI]: 1.42, 2.13). Bias estimates in the remaining income levels were substantially higher: positivity was 25.71 times (95% CI: 21.72, 30.09) and 24.38 times (95% CI: 21.29, 27.72) than that of surveillance data in UMICs and LMICs, respectively. The denial of new entry visas was associated with a relative risk reduction of 0.78 (95% CI: 0.72, 0.83) in UMICs. However, no significant effect was observed in LMICs (−0.13; 95% CI: −0.33, 0.03). Vaccination did not substantially alter post-arrival positivity. Conclusions: A comparison of entry screening data with local surveillance data indicated that ascertainment bias depends on income level.

## 1. Introduction

During the coronavirus disease 2019 (COVID-19) pandemic, infectious disease surveillance capacity was challenged in many countries given the overwhelming volume of infection cases [1]. Countries worldwide, including those recognized as having high-performance surveillance capacity (e.g., the United Kingdom), commonly experienced a substantial surge in cases [2,3]. As a policy response, many countries sought to directly strengthen capacity by expanding laboratory testing volumes, using digital technologies for monitoring and notification, or temporarily allocating human resources to corresponding public health sections [4,5]. In low-income countries where other infectious diseases continued to circulate during the COVID-19 pandemic, surveillance was adapted to accommodate the additional task of monitoring severe acute respiratory syndrome coronavirus 2 (SARS-CoV-2) [6]. Kenya, Malawi, and 14 other low- and middle-income countries (LMICs) modified diagnostic networks and information management systems for human immunodeficiency virus and tuberculosis to support COVID-19 testing [7].

Despite such efforts, the unavoidable introduction of ascertainment bias into surveillance data created a gap between the documented number and the actual number of infections. Ascertainment bias may be deconstructed into numerous components including virus pathogenicity, medical attendance-seeking behavior, motivation to test in a clinical setting, and strictness of notification. These components are characterized by a range of biological and societal factors, including the emergence of a new variant of concern, vaccine rollout, and a political shift toward prioritizing economic activity and the resumption of pre-pandemic social life [8,9,10,11,12]. Published studies have demonstrated the growing introduction of ascertainment bias into local surveillance data at both regional and national levels [13,14,15].

In this study, we investigated ascertainment bias by using airport entry screening data in Japan. In March 2020, the Japanese government implemented entry screening procedures that included testing of all passengers with real-time reverse transcription-polymerase chain reaction or antigen tests upon arrival; testing continued until early 2023. By contrasting the entry screening results with foreign travelers’ local surveillance data, this study evaluated the ascertainment bias in SARS-CoV-2 surveillance data in travelers’ countries of origin and stratified the bias by country income level.

## 2. Methods

### 2.1. Brief Description of the Border Control Policy in Japan

Japan implemented several border control measures, which were initially concentrated at three major international airports—Tokyo Narita, Tokyo Haneda, and Kansai—before gradually expanding to other airports. From March 2020 to December 2022, all foreign visitors were required to enter the country via those designated airports. All arriving passengers, including Japanese citizens, underwent entry screening with real-time reverse transcription-polymerase chain reaction (or antigen tests from late July 2020). Details of testing procedures can be found elsewhere [16]. From 1 September 2020, foreign visitors were additionally mandated to present a negative SARS-CoV-2 test certificate within 72 h of departure as part of exit screening before flying to Japan. This requirement was expanded to all travelers, including Japanese travelers, from 13 January 2021. A chronological list of border control events can be found elsewhere [17].

**Figure 1:**
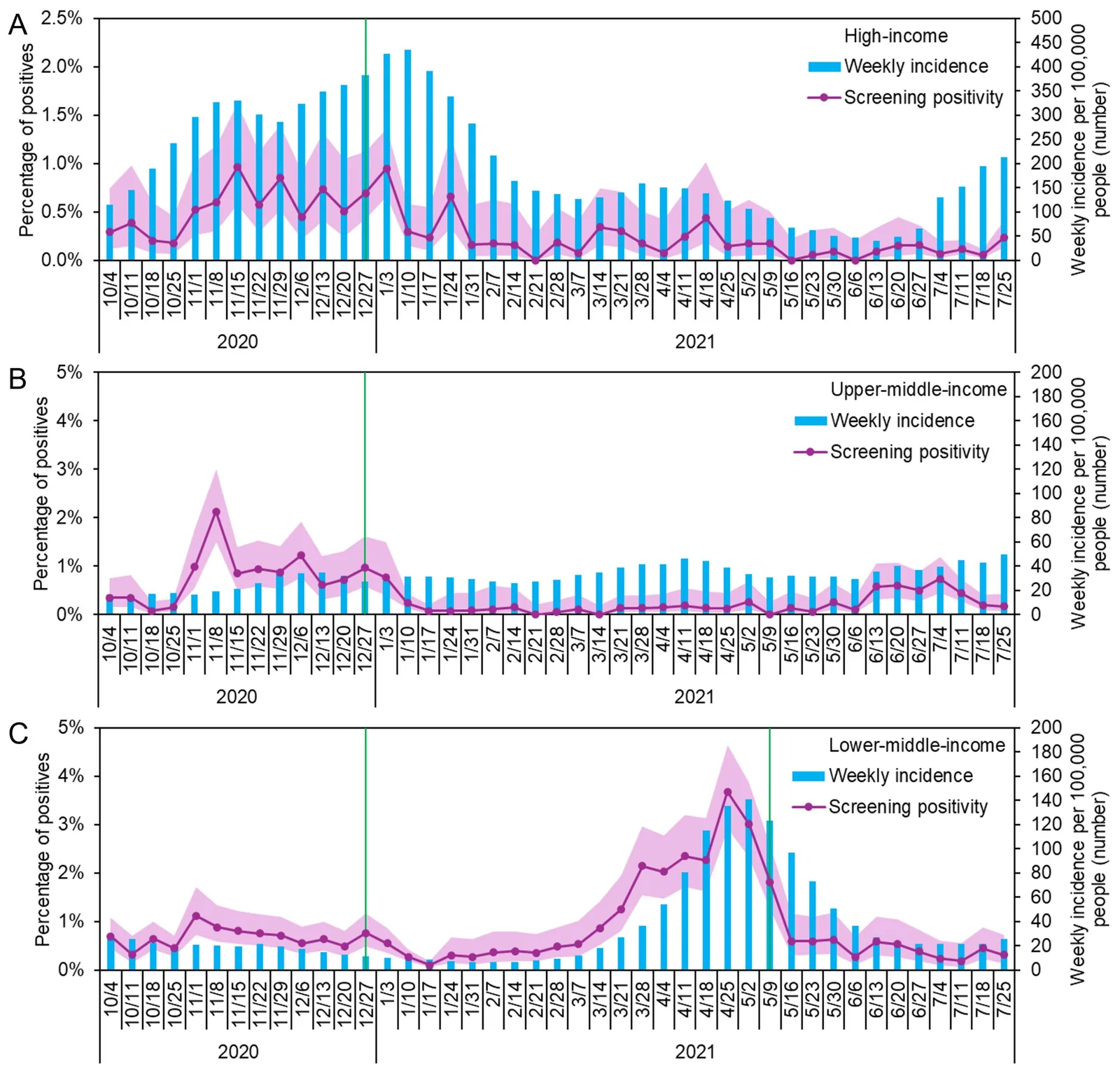
Weekly airport screening positivity among foreign travelers at the Japanese border vs. weekly SARS-CoV-2 incidence by income group, with relevant travel policies. The figure compares the weekly screening positivity rates at Japanese airports with the weekly severe acute respiratory syndrome coronavirus 2 incidence rates across income groups: (**A**) high income, (**B**) upper-middle income, and (**C**) lower-middle income. The purple dotted line with markers represents the weekly screening positivity rate among travelers, and the shaded purple area indicates the 95% confidence intervals. The blue bars show the weekly incidence per 100,000 people in the corresponding income groups. The first green line in each of the three panels represents the implementation of the denial of new entry policy on 28 December 2020. The second green line in (**C**) lower-middle income represents the implementation of the denial of re-entry policy on 14 May 2021.

In addition, Japan implemented traveler volume control policies that restricted entry and residence permits based on the infection risk assessment of incoming passengers. On 28 December 2020, new entry of foreign nationals from all countries was suspended (Figure 1). New entry refers to the permission granted to foreign nationals traveling to Japan for the first time under their current visa category. Re-entry—permitted for foreign nationals with residence permits in Japan, allowing them to return after temporary travel abroad—was suspended on 14 May 2021 for travelers from India, Nepal, and Pakistan (Figure 1) given the surge of the Delta variant (B.1.617.2) of SARS-CoV-2 in these countries [18]. During the suspension of new entries, re-entry and other visa categories such as family, diplomacy, and permanent residence continued to be permitted.

### 2.2. Epidemiological Datasets

The Ministry of Health, Labour, and Welfare publicly reported the results of entry screening from October 2020 to October 2022 [19] (Supplementary Data). The reports included weekly records of the total numbers of tested and positive cases categorized by nationality (i.e., Japanese and others). Detailed data regarding flight connections and specific nationalities among non-Japanese were unavailable. With a focus on the period from 4 October 2020 and 31 July 2021, this study considered only foreign passengers because most Japanese people stayed in the country of departure for a brief period and thus did not accurately reflect the risk of infection at the origin. To ensure estimation stability, countries with fewer than 30 travelers per week throughout the study period were excluded. No recorded positive cases or those with missing data—such as incomplete two-dose vaccination records—were excluded. Ultimately, 25 countries were included for further analysis (Table 1).

For each included country, the daily number of confirmed cases was retrieved from the repository of the Center for Systems Science and Engineering at Johns Hopkins University [20]. Additionally, datasets on two-dose vaccine records were obtained from Our World in Data [21], genome surveillance data of the variant of concern were derived from CoV-SPECTRUM [22], and information on population size was obtained from the Population Division of the Department of Economic and Social Affairs of the United Nations Secretariat [23].

### 2.3. Statistical Estimation by Income Group

For the statistical analysis, the 25 countries were first grouped by income level using the World Bank country classification [24]. The analysis was conducted by income level because surveillance and diagnostic testing capacities in the global health context are known to be dependent on a country’s income level [25,26,27,28]. Nine countries, including the United States, South Korea, the United Kingdom, France, Germany, Spain, Italy, Canada, and the United Arab Emirates, were classified as high-income countries (HICs). The eight upper-middle-income countries (UMICs) were China, Indonesia, Brazil, Russia, Thailand, Malaysia, Turkey, and Peru. The eight lower-middle-income countries (LMICs) were the Philippines, Vietnam, India, Nepal, Pakistan, Bangladesh, Ukraine, and Sri Lanka (Table 1).

Aggregated weekly airport screening positivity for each income group was calculated by dividing the total weekly number of positive travelers by the total weekly number of travelers (Figure 1). We also retrieved surveillance data from each country, and using the dataset, the daily prevalence was computed by taking a convolution of daily incidence and survivorship function of virus positivity derived from Dataset 2 in our earlier study [29], which was based on published estimate of positivity as a function of time since illness onset. The same distribution was applied throughout the study period. After estimating a 7-day average of daily prevalence, the weekly prevalence of each income group was weighted by population size.

Travelers arriving at airports in Japan were assumed to be a random sampling of the general population of the country of origin. Of course, this assumption does not fully hold and especially, sick travelers may cancel the travel; thus, the assumption provides a useful simplifying framework, but it should be remembered that the diagnosed proportion may be greater than what we report here. As such, the entry screening results among travelers were interpreted as a binomial sampling process in each week. The positivity rate $s_{i}(t)$ in airport screening was considered proportional to the surveillance-based prevalence estimate $p_{i}(t)$ in week t, where i represented the income group index (i = HICs, UMICs, and LMICs) and t represented the week starting from 4 October 2020.

To estimate the country-level ascertainment bias in an income group i, a constant parameter $\alpha_{i}$ was multiplied by the surveillance-based prevalence to fill the gap in infection risk between the airport positivity rate and the surveillance-based prevalence. This relationship was modeled as a piecewise function, with breakpoints representing either the timing of an intervention measure or the decision and implementation of a new policy. We modeled the airport positivity airport $s_{i}(t)$ as

$$s_{i}\left(t\right)=\{\begin{array}{l} \alpha_{i}p_{i}\left(t\right)\;\;\;\;\;\;\;\;\;\;\;\;\;\;\;\;\;\;\;\;\;\;\;\;\;\;\;\;\;\;\;\;\;\;\;\;\;\;\;\;\;\;\;\;\;\;\;\;\;\;\;\;\;\;\;for\;t<A_{i}\;\;\;\;\;\\ \alpha_{i}p_{i}\left(t\right)\left(1-vf_{i}(t)\right)\;\;\;\;\;\;\;\;\;\;\;\;\;\;\;\;\;\;\;\;\;\;\;\;\;\;\;\;\;\;for\;A_{i}\leq t<B_{i}\\ \alpha_{i}p_{i}(t)(1-vf_{i}(t))(1-n_{i})(1-m)\;\;\;\;\;for\;B_{i}\leq t<C_{i} \end{array},$$

where $p_{i}(t)$ was the estimated prevalence of group i. From time $A_{i}$, the equation incorporated v, which represented the public health impact of vaccination on travelers and may be interpreted as the vaccine-induced relative change in positivity among travelers, and $f_{i}(t)$ was the two-dose vaccination coverage of income group i. Notably, in addition to the biological protective effect of vaccination, v was influenced by the frequency of risky contact and mobility among vaccinated individuals and thus was not guaranteed to have a value less than 1. Moreover, ($1-vf_{i}(t))$ corresponded to the relative risk of infection among vaccinated versus unvaccinated travelers. Time $A_{i}$ corresponded to the week in which two-dose vaccination was initiated, i.e.,

$$A_{i}=\{\begin{array}{c} 11\;\mathrm{if}\;i=HICs\;(Week\;11=13\;Dec\;2020)\\ 17\;\mathrm{if}\;i=UMICs\;(Week\;17=24\;Jan\;2021)\\ 19\;\mathrm{if}\;i=LMICs\;(Week\;19=7\;Feb\;2021) \end{array}.$$

By the end of the study period on 31 July 2021, two-dose vaccination coverage had reached 50% in HICs, 33% in UMICs, and 6% in LMICs. Times $B_{i}$ and $C_{i}$ corresponded to the week in which the ban on new entry of foreign nationals was canceled and the last week of the study period, respectively, resulting in $B_{i}=13\left(Week\;13=27\;Dec\;2020\right)$ to $C_{i}=43\left(Week\;43=25\;Jul\;2021\right)$ for all income groups. When new entry was sharply denied, we assumed that the relative risk reduction was $n_{i}$. Additionally, on 14 May 2021, re-entry for travelers from India, Nepal, Pakistan, and later Bangladesh [30] was suspended to maximize safeguards against the Delta variant. The relative risk reduction, m, induced by the prohibition of re-entry was considered only in LMICs. Thus, the relative risk of infection under denial of re-entry compared with that in the pre-denial period was (1−m).

Although not explicitly described, we tested various alternatives (e.g., vaccine effects by income group i and the emergence of a variant of concern) to bridge the gap between airport positivity and estimated local prevalence. By exploring all possible combinations (i.e., adding or not accounting for such additional factors), we identified the best fit model by comparing the Akaike information criterion (AIC).

Maximum likelihood method was used to estimate unknown parameters. The likelihood in one income group was considered conditionally independent from that of other income groups, and the total log likelihood was obtained by summing the individual log likelihoods across all income groups. The 95% confidence intervals (CIs) of the parameters were determined by using the profile likelihood. To calculate the 95% CIs of the model predictions, a parametric bootstrap method was used to resample parameters and generate model predictions 10,000 times. Software used included JMP Pro version 17.0.0 (SAS Institute Inc., Cary, NC, USA) and Microsoft Excel version 2024 (Microsoft Corporation, Redmond, WA, USA).

### 2.4. Statistical Estimation by Country

Although the group unit in this study was determined by income level, we also explored country-specific result for countries, including India, Nepal, and Pakistan, with a particularly high prevalence of COVID-19 during the study period. These three countries were geographically close, non-zero COVID-19 countries and were subject to the re-entry denial policy implemented on 14 May 2021.

## 3. Results

### 3.1. Screening Positivity vs. Incidence

Figure 1 presents the weekly screening positivity at Japanese airports and weekly local incidence of COVID-19 by income group overlaid with relevant travel policies. Before the denial of new entry (indicated by first vertical green line) on 28 December 2020, airport screening positivity showed an upward trend in HICs and UMICs, mirroring local incidence at origin. After policy implementation, screening positivity began to decline, although incidence continued to plateau or rise in HICs and UMICs. These positivity and local incidence trends were not visually identifiable in LMICs. Additionally, after denial of re-entry for travelers from India, Nepal, Pakistan, and later Bangladesh from 14 May 2021, both incidence and screening positivity in LMICs declined.

Table 1 presents the total number of incoming travelers in 2020 and 2021 categorized by country and income level. LMICs represented the highest total traveler volume of 102,318 travelers, of whom 743 tested positive, resulting in the highest positivity of 0.73% (95% CI: 0.68, 0.78). HICs followed by volume of travelers, with 94,848 travelers and 243 positive cases, representing the lowest positivity of 0.26% (95% CI: 0.23, 0.29). UMICs recorded 83,699 travelers with 290 positive cases, yielding a positivity of 0.35% (95% CI: 0.31, 0.39).

### 3.2. Parameter Estimates by Income Level

**Figure 2:**
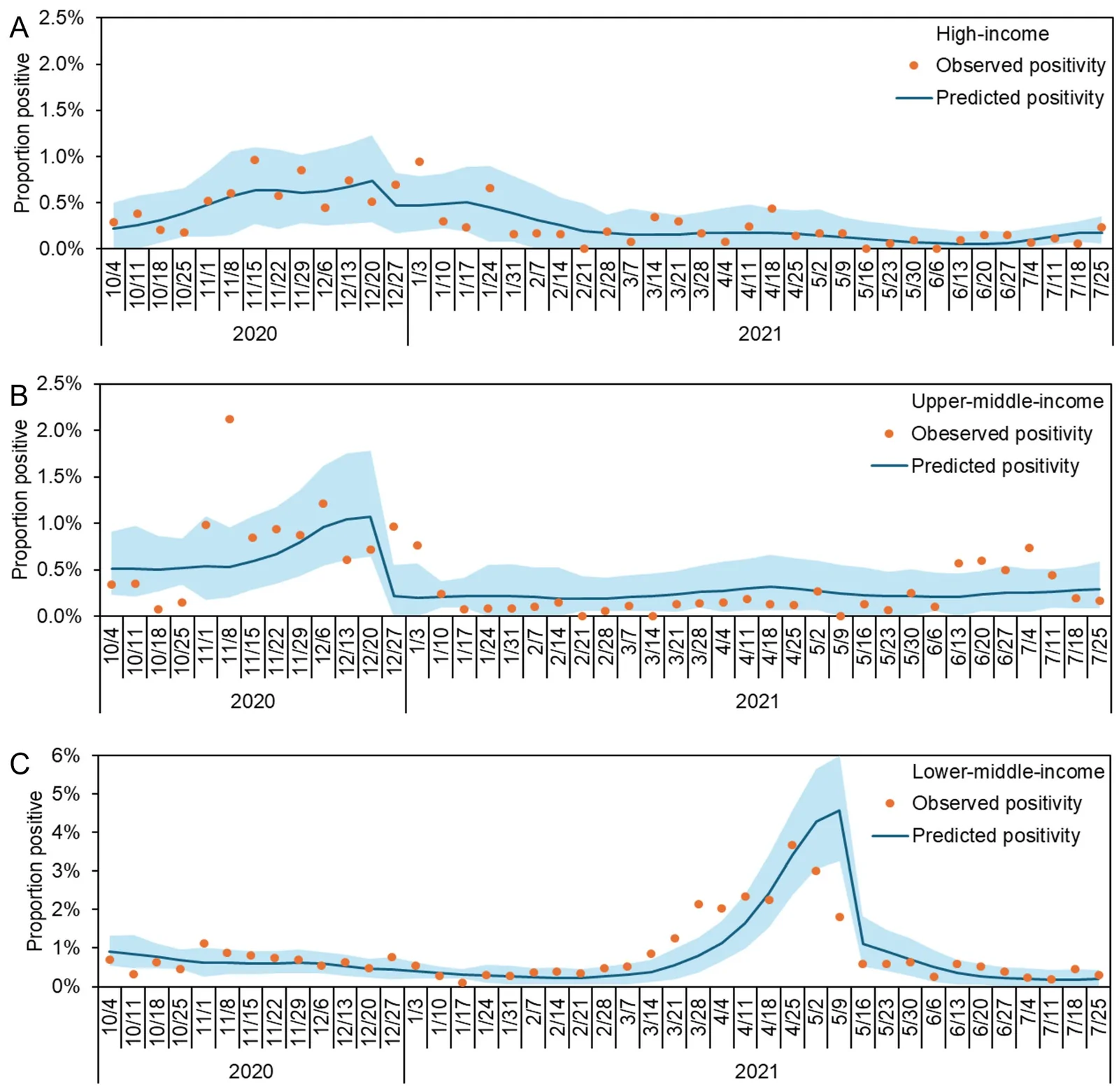
. Comparison of observed and predicted positivity across income groups from 4 October 2020 to 31 July 2021. The figure compares the observed and predicted positivity from airport screening among travelers across income groups. Panels represent the three income groups: (**A**) high income, (**B**) upper-middle income, and (**C**) lower-middle income. The orange dots represent the observed proportion of positive cases detected through airport screening. The blue line indicates the predicted positivity for each income group, and the shaded blue area denotes the 95% confidence intervals, which were calculated using the bootstrap method.

Table 2 presents the model’s parameter estimates by income group. The ascertainment bias in HICs was the lowest, with a value of 1.76 (95% CI: 1.42, 2.13) times the estimated local incidence. In contrast, the ascertainment bias was estimated at 25.71 (95% CI: 21.72, 30.09)- and 24.38 (95% CI: 21.29, 27.72)-times higher than the local incidence in UMICs and LMICs, respectively—substantially higher than in HICs. Although both UMICs and LMICs showed greater bias estimates compared with HICs, the difference between these two groups was relatively small. The denial of new entry visas was associated with the greatest relative risk reduction in UMICs, with an estimate of 0.78 (95% CI: 0.72, 0.83), followed by HICs at 0.41 (95% CI: 0.22, 0.56). In contrast, no clear risk reduction was associated with this policy in LMICs, with an estimate of −0.13 (95% CI: −0.33, 0.03). The denial of re-entry for travelers from India, Nepal, Pakistan, and later Bangladesh was associated with a relative risk reduction of 0.74 (95% CI: 0.67, 0.80) in LMICs. The estimated vaccine coefficient was 0.22 (95% CI: −0.54, 0.80). The wide CI indicated substantial uncertainty; the range spanned both the risk reduction in positivity due to vaccination and the controversial increase in the risk of infection among travelers. Figure 2 illustrates the observed positivity from airport screening alongside predicted positivity from the model across income groups. The observed and predicted positivity shared close time-dependent patterns within each income group, demonstrating consistency between airport screening data and the model’s predictions.

### 3.3. Comparison of Models

Table 3 compares the results of the main model and those of two other models using AIC. Model 2 incorporated the impact of the variant of concern (i.e., the combined impact of Alpha and Delta variants) by multiplying a constant that was weighted by the proportion of each variant based on genomic surveillance data [22]. However, the AIC value of Model 2 was higher than that of the main model. Model 3 introduced differing vaccine effects for each income group. Although this model yielded a slightly lower AIC compared with the main model, the model estimates were counterintuitive. Specifically, the coefficient estimate of two-dose vaccination coverage and its impact on relative change in positivity by income group were not sensible (i.e., the UMIC estimate had a negative value and the HIC estimate was far lower than that of LMICs, despite LMICs having the lowest vaccination coverage). Thus, Model 3 was excluded. The parameter estimates from Model 3 are provided in Supplementary Table S1.

### 3.4. Individual Country Estimates

In the analysis by South Asian country, the ascertainment bias estimates were over 30, 26 and 177 in India, Nepal and Pakistan, respectively. The analysis of India, Nepal, and Pakistan revealed that the policy of denying new entry did not positively influence the positivity at airports in Japan. However, denial of re-entry resulted in a relative risk reduction of 0.49 (95% CI: 0.18, 0.69) with substantially decreased traveler volume in the three countries (see Supplementary Table S2). Supplementary Figure S1 provides a comparison between the observed and predicted positive proportion in the three countries.

## 4. Discussion

This study evaluated the ascertainment bias of SARS-CoV-2 by comparing Japan’s airport entry screening data with surveillance data of the country of origin by country income level. The overall trends in the two datasets were generally shared across HICs and UMICs. Thus, we exploited the relationship between the datasets to estimate the ascertainment bias and other parameters that govern the intervention-associated risk modifications among travelers arriving in Japan. The ascertainment bias was lowest in HICs (i.e., 1.76 times the local prevalence). The estimates were substantially higher in UMICs (25.71 times) and LMICs (24.38 times) than in HICs. Denial of new entry resulted in the highest relative risk reduction of 0.78 (95% CI: 0.72, 0.83) in UMICs, followed by HICs at 0.41 (95% CI: 0.22, 0.56), whereas no significant effect was observed in LMICs (−0.13; 95% CI: −0.33, 0.03).

As an important take-home message, we demonstrated that airport screening data, especially when obtained through a universal testing policy, can help reconstruct COVID-19 epidemiology worldwide and elucidate the extent of ascertainment bias. During the pandemic, most HICs and some UMICs rapidly scaled up surveillance by investing in infrastructure, supplies, and medical personnel and strengthened testing and reporting capacity [31,32]. Although HICs had the highest average “reported” incidence, we showed that travelers from HICs exhibited the lowest positivity at airports and indicated the lowest ascertainment bias (1.76) among the three income groups. In contrast, many LMICs faced competing health priorities as existing surveillance systems were adapted for SARS-CoV-2 amid co-circulating infectious diseases [6]. For instance, stringent interventions in LMICs led to failure to report infections to surveillance in these countries. Moreover, we previously showed that the effectiveness of exit screening was lowest among LMICs [17]. Depending on countries’ income level and associated intervention policies, we characterized ascertainment effort. Our results indicated that an adjustment of ascertainment bias could be imposed in real time to local surveillance data by examining entry screening data. The finding that surveillance and testing capacities are different across income levels aligns with results of previous studies [25,26,27].

The denial of new entry visas was not to be effective in protecting against the arrival of infected individuals to Japan. Possible explanations are (i) intensified epidemic in the country of origin and (ii) substantial risk of infection among those with re-entry visas. First, restricting travelers to re-entry visa holders might not have been a good idea given the risk of infection in origin countries that were experiencing an intense epidemic. Substantial under-ascertainment likely fueled the risk of infection even among all visa holders, contributing to high positivity during entry screening. Second, the early emergence of the Delta variant in India and neighboring countries likely increased the risk of infection among travelers [29], and the overwhelming case load demand in origin countries may have increased the outbound mobility of re-entry visa holders. Moreover, the limited effectiveness of exit screening among travelers from LMICs was well known [17].

The model did not show a prominent relative change induced by vaccination, suggesting that the vaccine passport policy did not identifiably elevate the risk of infection among travelers. This finding does not directly refute the limited biological effectiveness of vaccination; rather, the overall policy’s impact in preventing positivity at airports was likely limited (and even further, absence of increased risk of infection under vaccine passport policy is an encouraging signal for the policy itself). Although we do not have explicit evidence, the possibility that vaccinated people have a greater number of contacts and travel opportunities than non-vaccinated individuals suggests that the risk of infection among vaccinated people was not substantially reduced compared with that in the pre-vaccination period. The present data do not allow us to directly test this possibility. This explanation could be regarded as a hypothesis requiring further investigation. Consistent with the effect observed here, a modeling study indicated that vaccination passports did not alter the positivity of airport screening [33]. In this study, although integrated with other interventions such as exit screening, vaccination passports did not have an observable effect in altering the overall risk of infection among travelers.

Three technical limitations must be discussed. First, international travel must have naturally selected healthy individuals to fly to Japan, resulting in lower observed positivity at airports compared with local prevalence at the origin [34]. Second, demographic differences, such as the age structure, between general and traveler populations may have introduced bias. Travelers tend to be young and healthy; however, local infections affect a broader age range of individuals with diverse backgrounds [35]. Because of the bias, our estimated ascertainment bias should be interpreted as a crude estimate rather than precise measurement at the population-level. Third, heterogeneous transmission dynamics (e.g., in countries with and without zero-COVID-19 policies) within UMICs did not allow the simple interpretation and generalization of results. For zero-COVID-19 countries including mainland China, very few positive travelers were identified at entry screening at Japanese airports, and ongoing transmission in these countries may have been geographically localized, such that entry screening may not have provided an accurate representation of local epidemiological dynamics. Therefore, aggregation at the UMIC level may have masked differences in the ascertainment bias between zero- and non-zero-COVID-19 countries.

## 5. Conclusions

Despite these limitations, we strongly believe that this study successfully characterized the relationship between airport entry screening data in Japan and travelers’ local surveillance data. Ascertainment bias can be estimated in real time using universal testing data, potentially helping reconstruct epidemiological dynamics worldwide.

## Figures and Tables

**Table 1:** Traveler volume by country and income category, including total travelers, SARS-CoV-2 positives, and positivity from 4 October 2020 to 31 July 2021.

Income Category	High-Income Countries		Upper-Middle-Income Countries		Lower-Middle-Income Countries	
Number of travelers from each country	United States of America	45,537	China	42,962	Philippines	46,107
	South Korea	18,218				
	United Kingdom	6601	Indonesia	15,725	Vietnam	22,580
	France	6576	Brazil	7749	India	11,911
	Germany	5892	Russia	5919	Nepal	10,109
	Spain	3528	Thailand	4690	Pakistan	5303
	Italy	3448	Malaysia	2974	Bangladesh	2638
	Canada	2606	Turkey	2070	Ukraine	2275
	United Arab Emirates	2442	Peru	1610	Sri Lanka	1395
Total number of travelers	94,848		83,699		102,318	
Total number of positives	243		290		743	
Positivity (95% CI)	0.26% (0.23, 0.29)		0.35% (0.31, 0.39)		0.73% (0.68, 0.78)	

**Table 2:** Parameter estimates of the model for entry screening results for COVID-19 at Japanese airports.

	Estimates (95% CI)
Ascertainment bias in HICs *	1.76 (1.42, 2.13)
Ascertainment bias in UMICs *	25.71 (21.72, 30.09)
Ascertainment bias in LMICs *	24.38 (21.29, 27.72)
RRR ^#^ from denial of new entry for HICs travelers	0.41 (0.22, 0.56)
RRR from denial of new entry for UMICs travelers	0.78 (0.72, 0.83)
RRR from denial of new entry for LMICs travelers	−0.13 (−0.33, 0.03)
Vaccine effect (coefficient of coverage)	0.22 (−0.54, 0.80)
RRR from denial of re-entry for LMICs ^$^ travelers	0.74 (0.67, 0.80)
AIC ^$^	15,511

* HICs: high-income countries; UMICs: upper-middle income countries; LMICs: lower-middle income countries. ^#^ RRR: relative risk reduction. ^$^: Specifically referring to travelers from India, Nepal, Pakistan and later Bangladesh. ^$^ AIC: Akaike Information Criterion.

**Table 3:** Model comparison for the test positivity among travelers arriving at Japanese airports in 2020–21.

No	Number of Parameters	Model	Akaike’s Information Criterion Value
1	8	Main model	15,511
2	9	Main model multiplies variant effect parameter with genomic proportion	16,102
3	10	Main model with 3 distinct vaccine effect in each income group	15,490

## Data Availability

Airport entry screening data for non-Japanese passengers are publicly available on the website of the Ministry of Health, Labour and Welfare [19] and are presented in the Supplementary File. The data sources are acknowledged for their contributions, and we adhered to their respective data-sharing policies and terms of use.

## Supplementary Materials

The following supporting information can be downloaded at: https://www.mdpi.com/article/10.3390/idr18050117/s1. Table S1: Parameter estimates of Model 3 for entry screening results for COVID-19 at Japanese airports; Table S2: Model parameter estimates for entry screening results in Japan among travelers arriving from India, Nepal, and Pakistan, 2020–21; Figure S1: Comparison of observed and predicted positivity in India, Nepal, and Pakistan; Supplementary Data: Weekly entry screening results.

## Abbreviations

CI	confidence interval
COVID-19	coronavirus disease 2019
HICs	High-income countries
UMICs	Upper-middle-income countries
LMICs	Lower-middle-income countries
SARS-CoV-2	severe acute respiratory syndrome coronavirus 2
