# Supplementary material for: Evaluating the Ascertainment Bias of SARS-CoV-2 Using Spatial Backcalculation of Airport Screening Data in Japan
Source: Infect Dis Rep. 2026 Oct 9;18(5):117. doi: 10.3390/idr18050117 (PMC13704272; doi:10.3390/idr18050117)

Supplementary Table S1. Parameter estimates of Model 3 for entry screening results for COVID-19 at Japanese airports.

	Estimates (95% CI)
Ascertainment bias in HICs*	1.76 (1.42, 2.13)
Ascertainment bias in UMICs*	25.71 (21.72, 30.09)
Ascertainment bias in LMICs*	24.38 (21.29, 27.72)
RRR[#] from denial of new entry for HICs travelers	0.33 (0.12, 0.50)
RRR from denial of new entry for UMICs travelers	0.84 (0.78, 0.88)
RRR from denial of new entry for LMICs travelers	-0.26 (-0.50, -0.07)
Vaccine effect (coefficient of coverage) in HICs	0.75 (0.10, 1.21)
Vaccine effect (coefficient of coverage) in UMICs	-3.15 (-6.27, -0.98)
Vaccine effect (coefficient of coverage) in LMICs	10.17 (4.46, 13.97)
RRR from denial of re-entry for LMICs[$] travelers	0.67 (0.56, 0.75)
AIC	15490

*HICs: High-income countries; UMICs: Upper-middle-income countries; LMICs: Lower-middle-income countries.

[#]RRR: Relative risk reduction.

$: Specifically referring to travelers from India, Nepal, Pakistan and later Bangladesh.

Supplementary Table S2. Model parameter estimates for entry screening results in Japan among travelers arriving from India, Nepal, and Pakistan, 2020–21

	India (95% CI)	Nepal (95% CI)	Pakistan (95% CI)
Ascertainment bias	30. 22 (21.42, 41.12)	25.72 (20.00, 32.42)	177.43 (110.93, 265.82)
RRR[#] from denial of new entry	-0.87 (-1.93, -0.21)	-2.40 (-3.70, -1.47)	-1.42 (-2.97, -0.56)
Vaccine effect (exponential)		22. 41 (7.01, 37.84)	
RRR from denial of re-entry		0.49 (0.18, 0.69)	

[#]RRR: Relative risk reduction.

Supplementary Figure S1. Comparison of observed and predicted positivity in India, Nepal, and Pakistan

This figure compares the observed and predicted positivity from airport screening for travelers in India, Nepal, and Pakistan. Panels represent (A) India, (B) Nepal, and (C) Pakistan. The orange dots represent the observed proportion of positive cases detected through airport screening. The blue line indicates the predicted positivity for each country, and the shaded blue area denotes the 95% confidence intervals, which were calculated using the bootstrap method. Missing observed positivity data points and confidence intervals indicate weeks with fewer than 30 travelers or no incoming travelers. Additionally, an exponential relationship for vaccine effect was considered for estimates for individual countries. The model yielded an Akaike information criterion that was extremely close to that of the main model, showing no evident improvement.

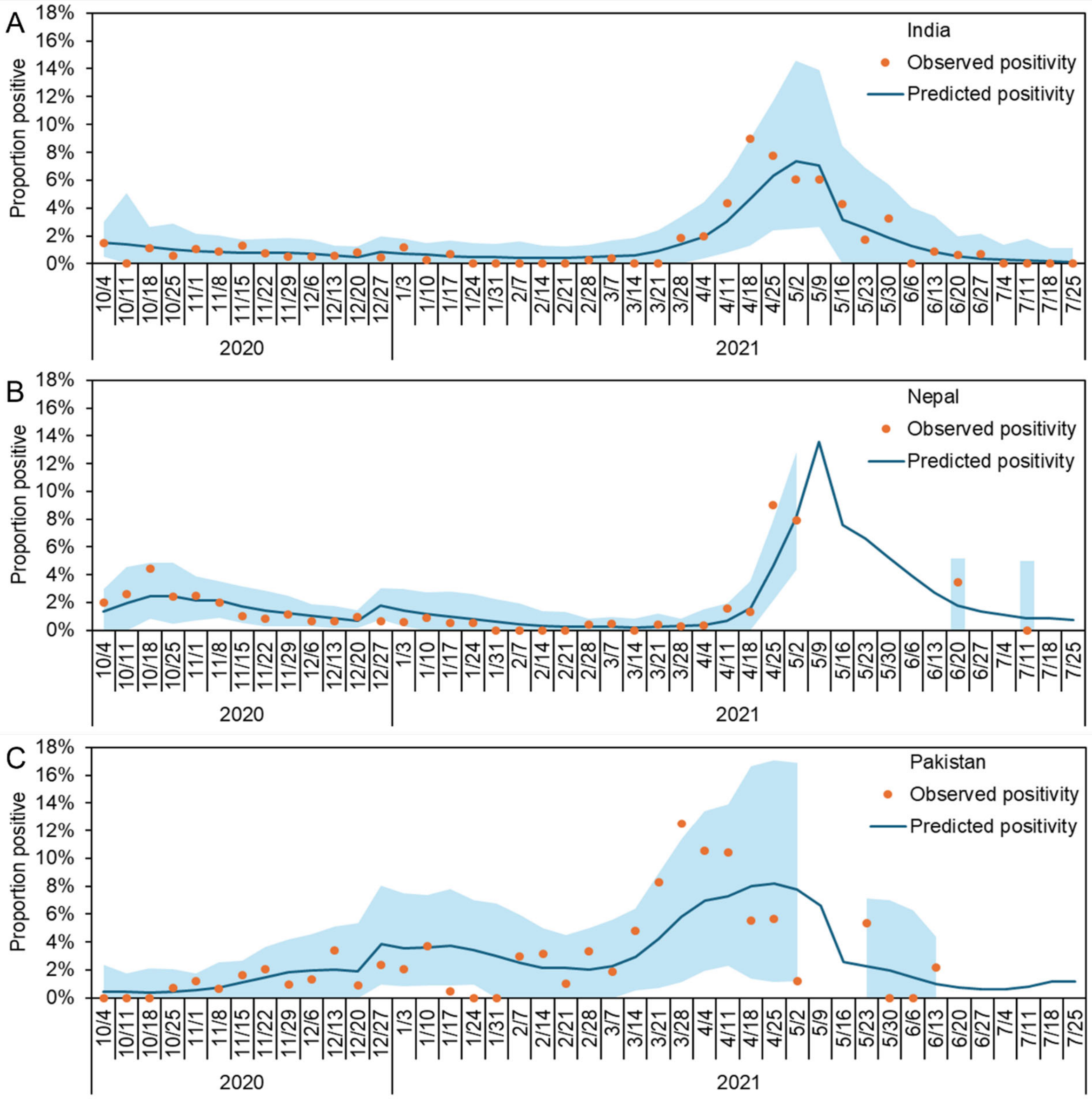

Supplement: Supplementary file 1 [file idr-18-00117-s001.zip › supplementary_tables and figure.pdf]
